# Mesoporous Silica Nanoparticles as Carriers for Intracellular Delivery of Nucleic Acids and Subsequent Therapeutic Applications

**DOI:** 10.3390/molecules22050782

**Published:** 2017-05-11

**Authors:** Wenzhang Cha, Rengen Fan, Yufeng Miao, Yong Zhou, Chenglin Qin, Xiangxiang Shan, Xinqiang Wan, Jinbo Li

**Affiliations:** 1 Department of General Surgery, Yancheng City No. 1 People’s Hospital, Yancheng 224001, China; Zhawenzhang@sohu.com (W.C.); fanrengen@sina.com (R.F.); zhouyong401@126.com (Y.Z.); taishanqinchenglin@163.com (C.Q.); 2 Department of Medical Oncology, Yancheng City No. 1 People’s Hospital, Yancheng 224001, China; miaoyufeng823@sohu.com; 3 Department of Gerontology, Yancheng City No. 1 People’s Hospital, Yancheng 224001, China; 4 Department of Clinical Medicine, Nantong University Xinglin College, Nantong 226000, China; 5 School of Chemistry and Chemical Engineering, Nanjing Unviersity, Nanjing 210023, China

**Keywords:** mesoporous silica nanoparticles, nucleic acids, drug delivery, therapeutics

## Abstract

Nucleic acids, including DNA, microRNA (miRNA), small interfering RNA (siRNA), and antisense oligonucleotide (ASO), are powerful gene regulators, which have been demonstrated as promising drug candidates for therapeutic treatments. Nevertheless, poor cellular membrane permeability and serum stability have greatly hindered the applications of nucleic acids in biomedicine. To address these issues, associate carriers that can encapsulate and protect nucleic acids are urgently required. Mesoporous silica nanoparticles (MSNs or MSNPs), which are nanomaterials with excellent biocompatibility, large surface area for functionalization, and tunable pore size for encapsulating different cargos, are emerging as novel and ideal biomaterials for different biomedical applications. In this review paper, we focus on the applications of MSNs in nucleic acid delivery and nucleic acid-guided therapeutic treatments. General strategies for the preparation of nucleic acid-MSN complexes will be firstly introduced, followed by a summary of recent applications of MSNs in nucleic acid delivery and nucleic acid-guided therapeutics.

## 1. Introduction

In the past two decades, nucleic acids, including DNA [1,2], microRNA (miRNA) [3,4], small-interfering RNA (siRNA) [5,6], and antisense oligonucleotide (ASO) [7,8], have attracted much attention for their tremendous potential in therapeutic applications. Generally, DNAs such as plasmid DNAs (pDNAs) work through re-expression of deficient gene products in diseased tissue. Endogenous miRNAs and exogenous siRNAs are able to recognize endogenous target mRNAs through sequence complementarity and regulate gene expression through repressing translation or inducing degradation, which are termed as RNA interference (RNAi) [9]. Noteworthily, endogenous miRNAs play critical roles in the occurrence and development of various human diseases including cancers [10,11], which has led to the rapid development of miRNA-targeted biomedicine with miRNA mimics or antisense miRNA oligonucleotides as pro-drugs [12]. ASOs, which are single-stranded nucleic acids and complementary to endogenous mRNAs, generally bind with and regulate misspliced mRNAs through inactivating them [13]. In light of their specific and powerful function in gene regulation, nucleic acids have been extensively investigated to explore their potential in therapeutic treatments and have also even entered clinic trials in recent years. For instance, pDNAs have been employed as potential vaccines in clinic trials [14]. MiR-34a mimics (5’-UGGCAGUGUCU-UAGCUGGUUGU-3’) [15] and ASO targeting miR-122 (5’-CCATTGTCACACTCC-3’) [16] have both been employed in clinics for treatment of cancer and hepatitis C virus (HCV), respectively. Several siRNAs have also been developed to treat renal and hepatic disorders [17]. These promising applications then greatly encouraged the development of different nucleic acids-guided therapeutic treatments.

Nevertheless, due to the negative charge of nucleic acids and poor stability in biofluids that prevent them from entering cells, efficient intracellular delivery of nucleic acids has emerged as the major obstacle for their further therapeutic applications. Current strategies for nucleic acid delivery mainly rely on the association of different carriers, such as virus vectors [18], lipid nanoparticles [19], and peptides [20], to overcome the inherent limitations. Ideal carriers for nucleic acid delivery should possess features such as easy synthesis and modification for introduction of different functionalities, easy loading of nucleic acids, excellent biocompatibility, high delivery efficiency, etc. Issues resulting with additional carriers during nucleic acid delivery, such as immune reactions caused by virus vectors, cytotoxicity induced by lipid nanoparticles, poor delivery efficiency of peptides, have led to an urgent need for alternative carriers. Solid nanoparticles, which have defined shape and size, are emerging as promising carriers for drug delivery [21,22]. The unique structures and large surface areas of nanoparticles allow efficient loading of different drugs, and the presence of chemical groups on the surface of nanoparticles also permits introduction of other functionalities, such as targeting ligands for target-selective delivery and fluorophores for tracing the delivery process. The biocompatibility and delivery efficiency of the nanoparticle carriers are then critical for successful nucleic acid delivery and therapeutics.

Different from heavy metal-based nanoparticles, mesoporous silica nanoparticles (MSNs or MSNPs) are silica-based nanostructured materials which exhibit excellent biocompatibility and chemical stability [23,24]. In comparison with other nanomaterials, MSNs possess unique features, such as large surface areas, unique porous structures, large pore volumes, and uniform and tunable pore size, which have received great attention in recent years for their use as biomaterials for various biomedical applications [25,26]. For examples, MSNs have been widely used in enzyme immobilization [27], tissue engineering [28], diagnostics [29,30], and delivery of diverse drugs including small-molecule and nucleic acid drugs [26,31,32,33]. In this review paper, we focus on the applications of MSNs in nucleic acid delivery and subsequent nucleic acid-guided therapeutics. We will first introduce the general strategies for the preparation of nucleic acid-MSN complexes, followed by a summary and discussion of their applications in intracellular delivery of nucleic acids and nucleic acid-guided therapeutic applications.

## 2. General Strategies for Preparation of Nucleic Acid-MSN Complexes

**Scheme 1:**
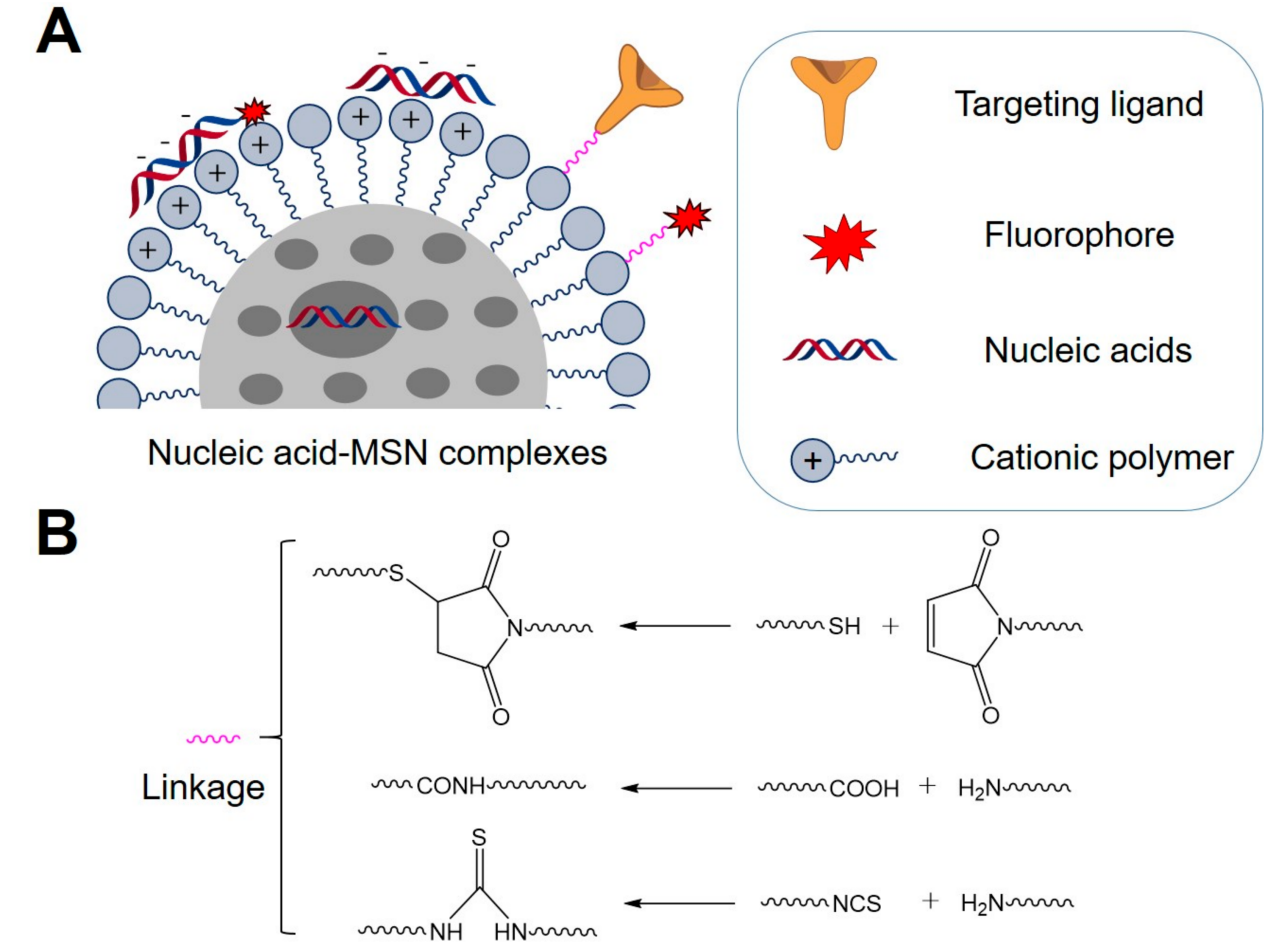
(**A**) Main structure and components of nucleic acid-MSN complexes; (**B**) Chemical strategies for conjugation of targeting ligands or fluorophores.

Since the strategies for synthesis of MSNs have been reviewed elsewhere [31,34], herein we mainly introduce the general strategy for preparation of nucleic acid-MSN complexes (Scheme 1). Non-covalent strategies were often used for preparation of nucleic acid-MSN complexes, due to the easy loading of nucleic acids onto MSNs and their quick release upon delivery into cells. As nucleic acids are negatively charged, MSNs are usually coated with cationic molecules on the surface to condense with nucleic acids through electrostatic and hydrophobic interactions (Scheme 1A). For example, amino molecules were used to functionalize the surface of MSNs for loading of pDNA [35,36]. Polyethyleneimine (PEI) coating on the surface of MSNs was used to package siRNA and DNA [37,38]. Polylysine and polyarginine were also coated on the surface of MSNs to condense siRNA [39,40]. Additionally, in comparison with other nanomaterials, the unique pore structure of MSNs also allows loading of different cargos inside the pores [24]. Therefore, it is possible to encapsulate small sized drugs inside the MSN pores to facilitate their delivery into cells [41]. However, due to the large size, nucleic acids usually could not be loaded into small pores. To expand the applications of MSNs for nucleic acid delivery, some research groups have also developed MSNs with large pores (>10 nm) that had enough capacity to encapsulate siRNAs (Scheme 1A) [39,42], which was shown to enhance the loading and delivery efficiency of nucleic acids. Moreover, the nucleic acid/MSN ratio of the complexes was usually optimized and determined through evaluation of their complexing and delivery efficiency of nucleic acids by MSNs.

Moreover, ideal functionalized MSNs for nucleic acid delivery should also contain other functionalities, such as targeting ligands and fluorophores. Targeting ligands can increase the active targeting of nucleic acid-MSN complexes and enhance the delivery efficiency of nucleic acids, while fluorophores can realize visualization of the delivery process and biodistribution of nucleic acids. These functionalities could be conjugated to MSNs through basic chemical reactions (Scheme 1B) [26,43], including thiol/maleimide coupling, condensation reactions between carboxylic acids and amines, or condensation reactions between amines and isothiocyanates. With these conjugation methods, the surface of MSNs have been modified with various targeting ligands or fluorophores for multi-functional theranostic applications. For instances, mannose that can bind to the mannose receptor that is highly expressed on the cellular membrane was attached onto the surface of MSNs through the reaction between amines and isothiocyanates, which resulted in functionalized MSNs for receptor-mediated delivery of DNAs [44]. Folic acid that can recognize folate receptor that is highly expressed on the membrane of many cancer cells was integrated onto MSNs through condensation reactions between carboxylic acids and amines, which allows selective delivery of siRNA into folate receptor positive HeLa cells [45]. The fluorophore FITC was also attached to MSNs through a co-condensation method involving the reaction between amines and isothiocyanates for tracing MSNs inside living cells [46]. For trafficking nucleic acids delivered by the nucleic acid-MSN complexes, nucleic acids are also usually labeled with fluorophores during synthesis for further investigation (Scheme 1B).

With these loading or modification strategies, diverse nucleic acid-MSN complexes with different functionalities were prepared for nucleic acid delivery and consequent nucleic acid-guided therapeutic applications. The intracellular delivery of nucleic acids mainly relies on the endocytosis of nucleic acid-MSN complexes into cells through non-specific or receptor-mediated cellular uptake, followed by escape of the complexes from the endosome/lysosome and release of nucleic acids from the complexes into the cytoplasm to exert their biological function [23].

## 3. MSNs as Carriers for Intracellular Delivery of Nucleic Acids and Nucleic Acids-Guided Therapeutic Applications 

Synthetic nucleic acids, such as DNA, miRNA, siRNA and ASO, are powerful in modulating endogenous gene expression. In light of their high specificity and relatively low toxicity, numerous gene-targeted therapeutics with synthetic nucleic acids as pro-drugs have been developed for treatment of diseases including cardiovascular disease [47], inflammation [48], infection [49], and cancers [50]. However, due to the negative charge of nucleic acids that prevents them from crossing the cellular membrane, intracellular delivery of nucleic acids still represents a big challenge in nucleic acid-guided therapeutic applications. With the development and employment of biocompatible MSNs as carriers, nucleic acids were well packaged for delivery into cells and regulating target gene expression to achieve therapeutics [25,51]. In this section, we will summarize and discuss the recent applications of MSNs in intracellular delivery of these nucleic acids and nucleic acid-guided therapeutics. The information about the nucleic acid-MSN delivery systems was summarized and listed in Table 1. The nucleic acids used were often chemically modified with fluorophores for further cellular studies.

### 3.1. DNA-MSN Complexes for DNA Delivery and DNA-Guided Therapeutics

**Figure 1:**
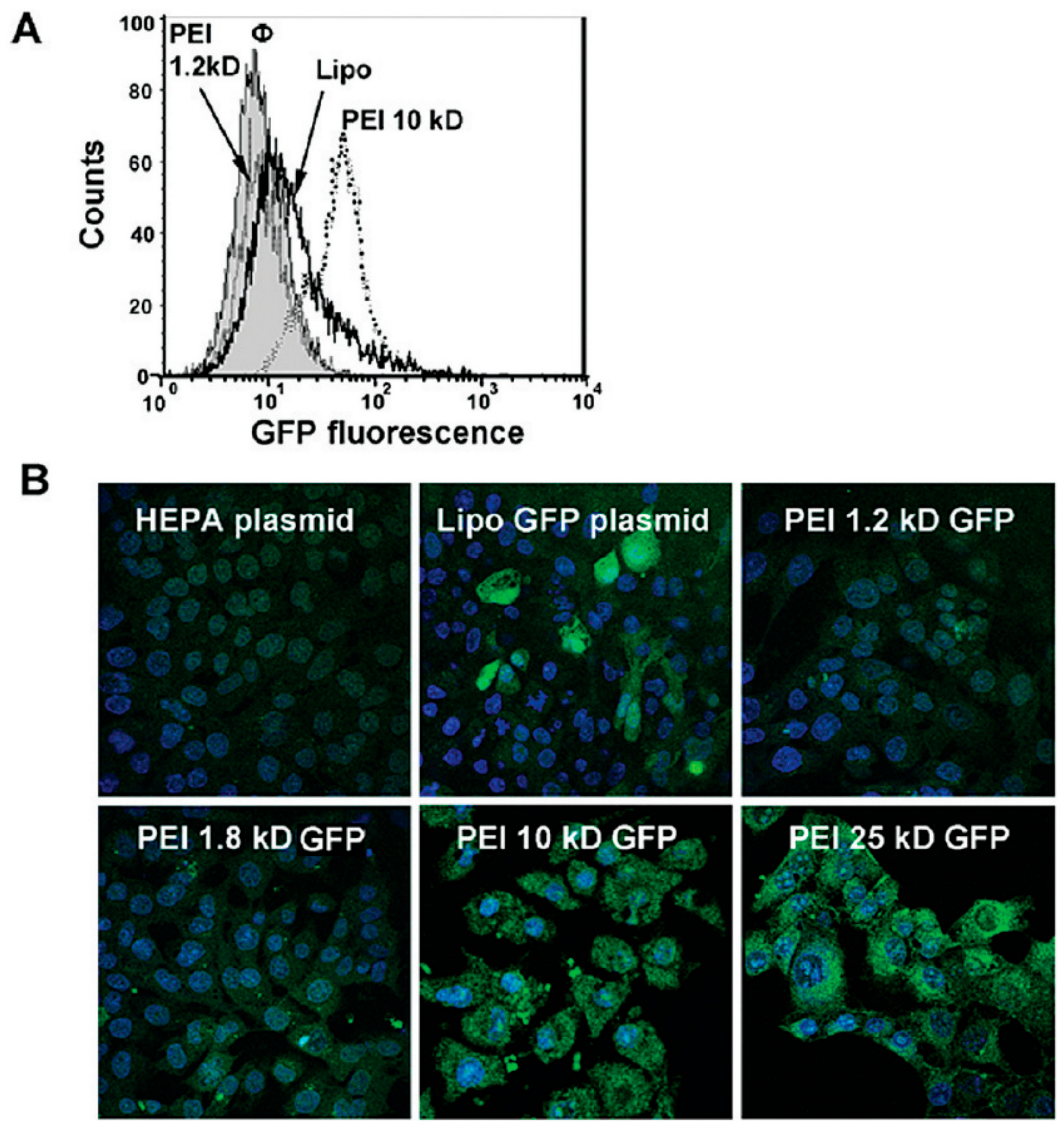
Transfection of GFP pDNA (100 ng/mL) into HEPA-1 cells with PEI-coated MSNs (25 μg/mL) or Lipofectamine as the carriers. The PEI size ranged from 1.2 kD to 25 kD as indicated in the figure. (**A**) Flow cytometry analysis of HEPA-1 cells; (**B**) Representative confocal images of HEPA-1 cells. Adapted from reference [37] with permission.

MSNs with different surface coatings that could complex with DNA through electrostatic interactions were often used for preparation of DNA-MSN complexes. Upon treatment of target cells with these DNA-MSN complexes, the complexes could be delivered into cells through endocytosis and DNA could be released from MSNs into the cytoplasm due to the change in microenvironment that destabilizes the DNA-MSN complexes. Meanwhile, in order to monitor and evaluate the intracellular delivery of DNA, reporter DNAs that are short DNAs labeled with fluorophores or pDNAs that could express fluorescent proteins were often used to prepare DNA-MSN complexes for further investigation. Through direct visualization of DNA delivery by detecting fluorescent signals, the delivery efficiency of DNA by different MSNs could thus be evaluated and compared. For examples, Vallet-Regi et al. used single-stranded DNA labeled with Cy3 as probe to examine the delivery efficiency of DNA by carbosilane dendron-decorated MSNs [52]. They found carbosilane dendron-decorated MSNs could efficiently mediate the internalization of DNA into a human osteoblast-like cell line without affecting the cellular viability. Using pDNA that could express into fluorescent mCherry protein, Gupta et al. also showed polyarginine coatings were effective in delivering pDNA into HeLa and A549 cells and the delivery efficiency by MSNs was even higher than that by commercial transfecting agent lipofectamine [40]. Different MSN surface coatings were shown to greatly affect the delivery efficiency of DNAs. To compare the effect of different surface coatings of MSNs on the delivery efficiency, Pichon et al. used pDNA that could express into luciferase to compare the delivery efficiency of histidine-functionalized MSNs with that of amine-functionalized MSNs [53]. In comparison with that of amine-functionalized MSNs, they found the delivery efficiency of pDNA by histidine-functionalized MSNs was significantly improved, as the fluorescent signals detected in cells treated with pDNA-histidine-MSNs were approximately two times stronger than those in cells treated with pDNA-amine-MSNs. The high delivery efficiency by histidine-functionalized MSNs was due to the ability of the imidazole moiety of histidine to destabilize membranes in an acidic environment, which subsequently promotes internalization and delivery of DNA into the cytoplasm. Moreover, Nel et al. also compared the size effect of PEI coatings of MSNs on the delivery efficiency of DNA [37]. They used pDNA that could express into green fluorescent protein (GFP) as the probe. Upon delivery of DNA by MSNs into cells, they compared the delivery efficiency through detecting GFP signals. As shown in Figure 1, the delivery efficiency by MSNs with 10 kD PEI was much higher than that by MSNs with 1.2~1.8 kD PEI and was even higher than commercial transfection agent lipofectamine. Even though MSNs with 25 kD PEI also showed high transfection efficiency, the toxicity was raised as another problem for intracellular delivery of pDNA, which suggested MSNs with 10 kD PEI was the ideal carrier for efficient delivery of DNA. These results indicate the surface modifications of MSNs are critical in MSN-mediated DNA delivery.

**Figure 2:**
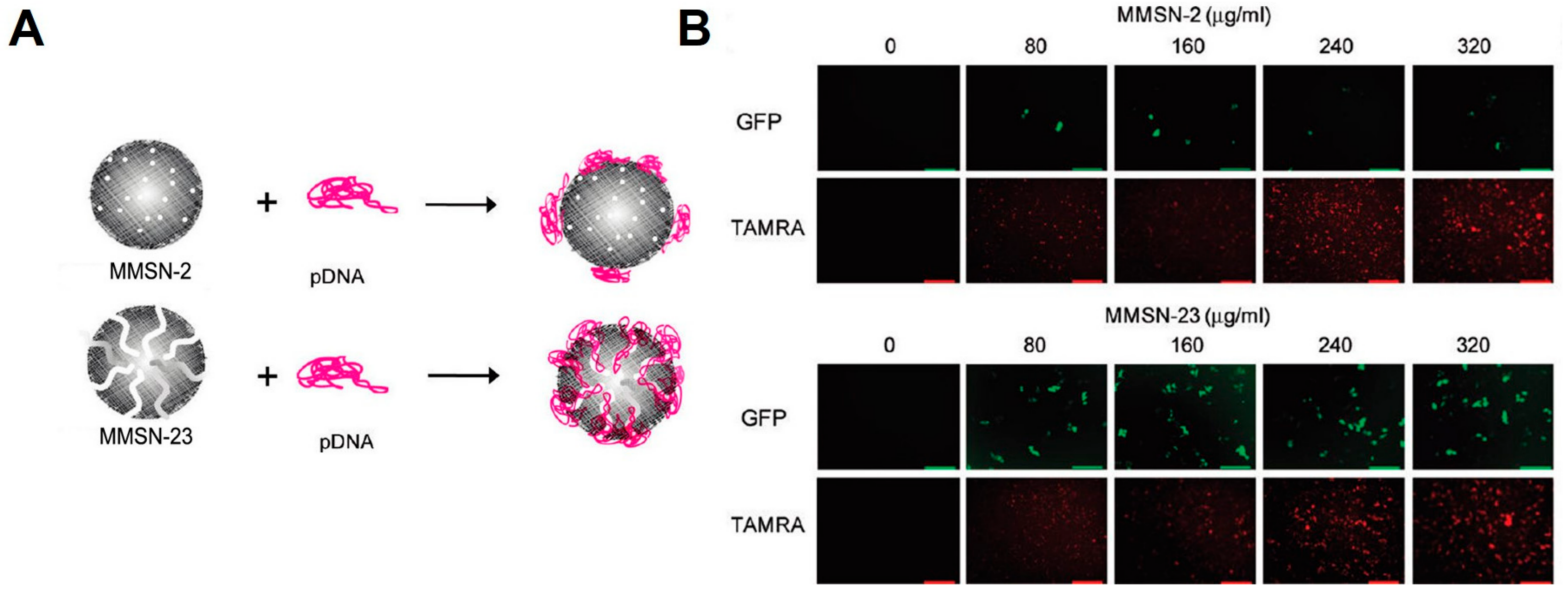
(**A**) Schematic illustration on preparation of DNA-MSN complexes with MMSN-2 or MMSN-23 as carriers; (**B**) Confocal fluorescent images of HeLa cells treated with pDNA(GFP)-MMSN-2 or pDNA(GFP)-MMSN-23. MMSNs were labeled with TAMRA. pDNA, 0.5 μg. Adapted from reference [55] with permission.

In addition to these examples using different surface coatings of MSNs to complex DNA for DNA delivery, other strategies aiming to enhance the delivery efficiency of DNA and biocompatibility of MSNs have also been developed. Rosenholm et al. added a cleavable linker, a disulfide bond, between MSNs and surface amine coatings [54]. DNA could thus be condensed onto the surface of MSNs through electrostatic interactions and efficient release of DNA could be triggered post cleavage of disulfide bond. By using amine-disulfide-modified MSNs as carriers, they found the release of DNA could be efficiently controlled through treatment with DTT or GSH that could break the disulfide bond. This strategy thus holds great promise in intracellular delivery of DNA into tumor cells, since reducing molecules are rich in the cytoplasm of tumor cells and could thus cleave the disulfide bond to realize efficient and controlled release of DNA. Additionally, the MSN pores have also been utilized to encapsulate DNA. Min et al. developed two types of amine-modified MSNs, monodispersed MSNs with small pores (~2 nm, MMSN-2) and monodispersed MSNs with large pores (~23 nm, MMSN-23) [55]. MMSN-2 was only able to condense DNA on the surface due to the small pore could not encapsulate DNA inside, while MMSN-23 could encapsulate DNA inside the large pore (Figure 2A). To compare the delivery efficiency of DNA by these two MSNs that encapsulate DNAs in two different ways, they used TAMRA to label MSN and used pDNA expressing GFP as probe. As shown in Figure 2B, the cellular uptake efficiency of MMSN-2 and MMSN-23 was comparable, since fluorescent signals from TAMRA detected in treated cells were comparable. While, as indicated by the GFP signals, delivery efficiency of DNA by MMSN-23 was quite higher than that by MMSN-2, indicating the utilization of large pore to encapsulate DNA could significantly improve the delivery efficiency.

**Figure 3:**
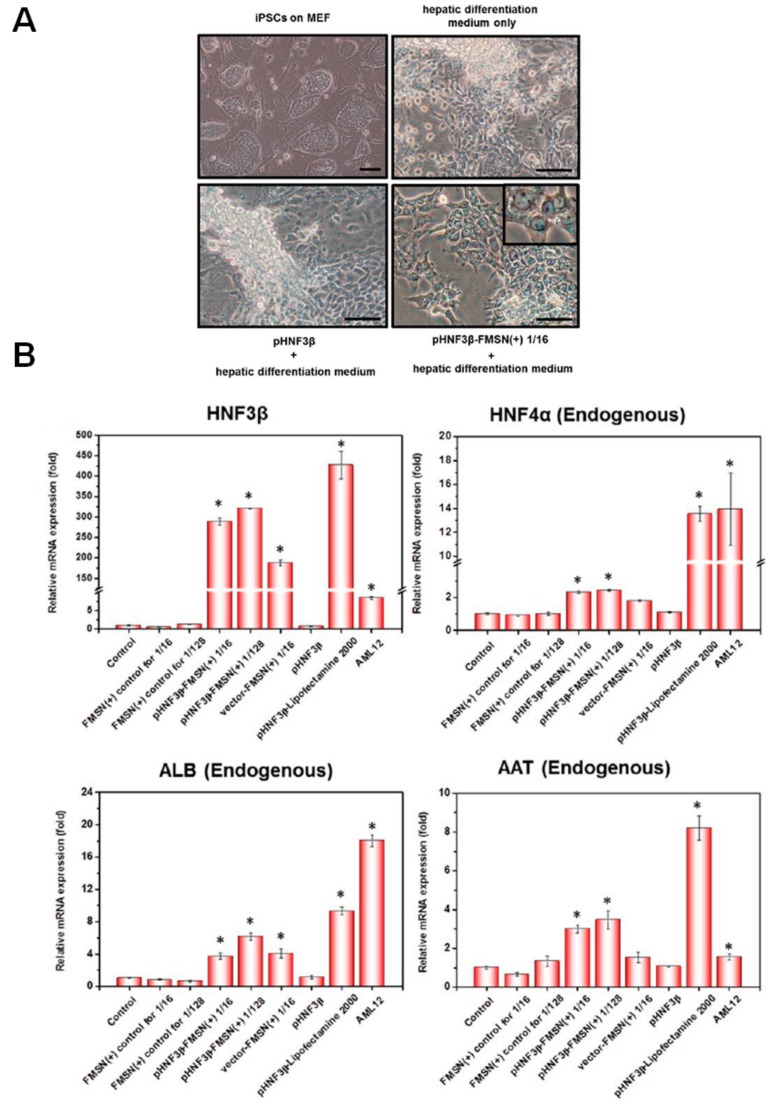
(**A**) Differentiation of iPSCs into hepatocyte like cells under hepatic differentiation medium supplemented with pHNF3β or pHNF3β-MSN (1/16 ratio) complexes; (**B**) Quantitative real-time PCR analysis of hepatocyte-specific markers, including HNF3β, HNF4α, ALB and AAT in iPSCs treated with control vector—MSN (1/16 or 1/28 ratio), pHNF3β-MSN (1/16 or 1/28 ratio), pHNF3β-Lipofectamine control, or other controls. pDNA, 1 μg/mL. Adapted from reference [46] with permission. * *p* < 0.05.

Through using different MSNs as carriers, DNAs could then be efficiently delivered into cells to exert their biological functions. For examples, immunostimulatory double-stranded DNA was delivered by MSN into Raw 264.7 cells [56]. Upon stimulation by the delivered DNA, type I interferon-α (IFN-α) was significantly activated. In comparison with treatment by naked DNAs (100 ng/mL), the concentration of IFN-α was increased from ~4 pg/mL to ~80 pg/mL post treatment by complexes that encapsulate same amount DNA with MSNs (DNA/MSN: 1/10 *w/w*), indicating the MSN-delivered DNA was still functional inside cells and warranting their further use for therapeutics. Inducing specific differentiation of stem cells is essential for personalized-regenerative medicine, which requires delivery of specific transcription factors into the stem cells. Mou et al. then used MSNs to deliver hepatocyte nuclear factor 3β (HNF3β) pDNA (1 μg/mL) that is a transcription factor for liver development into induced pluripotent stem cells (iPSCs) [46]. The expression of delivered HNF3β inside stem cells was able to induce their differentiation into hepatocyte-like cells (Figure 3A). Importantly, the MSN carriers did not modulate the endogenous levels of reactive oxygen species and pluripotent status, while mRNA expression levels of liver-specific genes were significantly enhanced by HNF3β (Figure 3B). Similarly, Kim et al. delivered bone morphogenetic protein-2 (BMP-2) pDNA (1 μg/mL, DNA/MSN: 1.85/10 *w/w*) that is a transcription factor for bone formation into mesenchymal stem cells (MSCs) by MSNs and the expression of delivered BMP-2 induced osteogenic differentiation of MSCs, which was further confirmed by detection of bone-related genes and proteins [57].

Since platelet derived growth factor (PDGF) is effective in healing, pDNA expressing PDGF (20 μg, DNA/MSN: 2/5 *w/w*) was then delivered into injured rat Achilles tendons by MSNs [58]. The expression of delivered PDGF then promoted healing of tendons and histological analyses suggested that tendons treated with pDNA-MSN complexes healed faster than untreated tendons or tendons treated with pDNA alone.

### 3.2. MiRNA/siRNA-MSN Complexes for miRNA/siRNA Delivery and miRNA/siRNA-Guided Therapeutics

**Figure 4:**
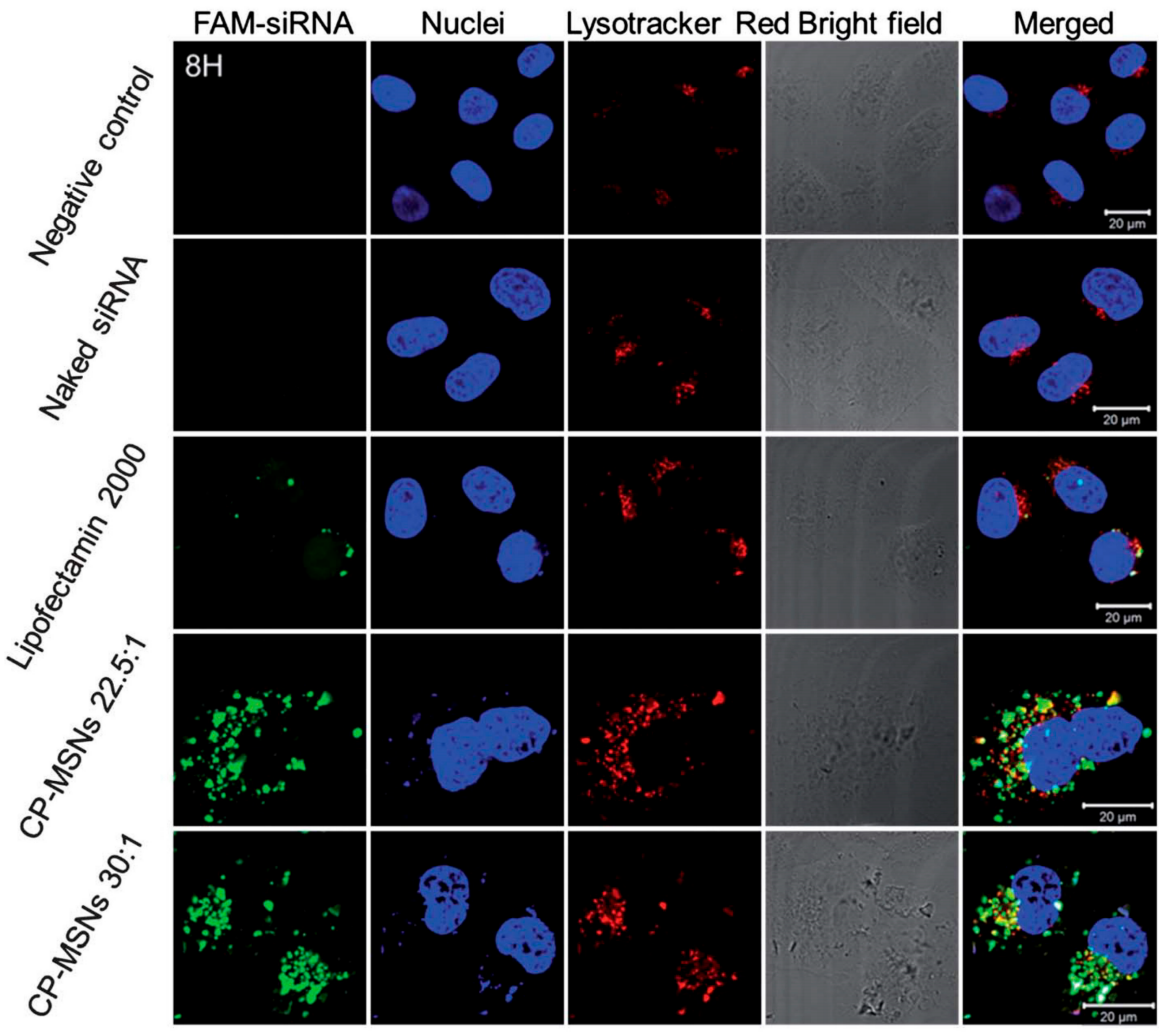
Trafficking of siRNA delivery into HeLa cells with CP-MSNs as carriers and FAM-labeled siRNA as probe (1 μg/mL). DAPI and Lysotracker was used to stain nucleus and lysosome, respectively. Adapted from reference [62] with permission.

MiRNAs and siRNAs are small non-coding RNAs with lengths of ~22 nucleotides, which hold great promise in gene therapy through suppressing gene expression at the post-transcriptional level. In order to prepare miRNA/siRNA-MSN complexes for intracellular delivery of miRNAs and siRNAs, self-assmebly strategies based on the electrostatic and hydrophobic interactions between negatively charged RNAs and positively charged surface coatings were often used to load miRNAs or siRNAs onto MSNs. Similarly as DNA delivery, miRNAs or siRNAs were delivered into cells through endocytosis of miRNA/siRNA-MSN complexes and then released from the complexes into cytoplasm. For examples, as reported by Dong et al. poly(2-dimethylaminoethyl methacrylate) (PDMAEMA)-modified MSNs (CP-MSNs) were used to condense siRNAs for siRNA delivery into HeLa cells [62]. To monitor the delivery, FAM-labeled siRNA was used as the probe. Upon treatment of HeLa cells with the siRNA-MSN complexes and further measurement of fluorescent signals form siRNA and stained lysosome, they found siRNA was also detected in the lysosome, indicating the delivery of siRNA was through endocytosis pathway. Noteworthily, the delivery efficiency by MSNs was even higher than that by commercial transfection agent lipofectamine (Figure 4).

Furthermore, to assess whether the siRNA delivered by MSNs was functional, siRNA against Lamin A/C was used and they found siRNA (1 μg/mL, siRNA/MSN: 1/30 *w*/*w*) delivered by MSNs was able to down-regulate expression levels of Lamin A/C. Similarly, Tamanoi et al. used PEI-coated MSNs to condense siRNAs targeting K-ras and they found MSNs could facilitate the delivery of siRNAs (7 μg/mL, siRNA/MSN: 1/25 *w*/*w*) into PNAC-1 cells to down-regulate the protein levels of K-ras, as well as p-Erk that is the downstream target of K-ras [63]. In addition to these examples using cationic polymers coated on MSNs to complex siRNAs, it is worth noting a novel non-covalent self-assembly strategy for miRNA delivery reported by He et al., which utilized a bi-functional peptide [60]. The bi-functional peptide consisted of two parts, a silica binding part (RGRRRRLSCRLL) and a miRNA binding part (KKKKKKKK). Due to the ion pairing, hydrogen bonding and van-der-Waals interactions, the silica binding peptide (RGRRRRLSCRLL) has been demonstrated to have strong binding with silica [74].

**Figure 5:**
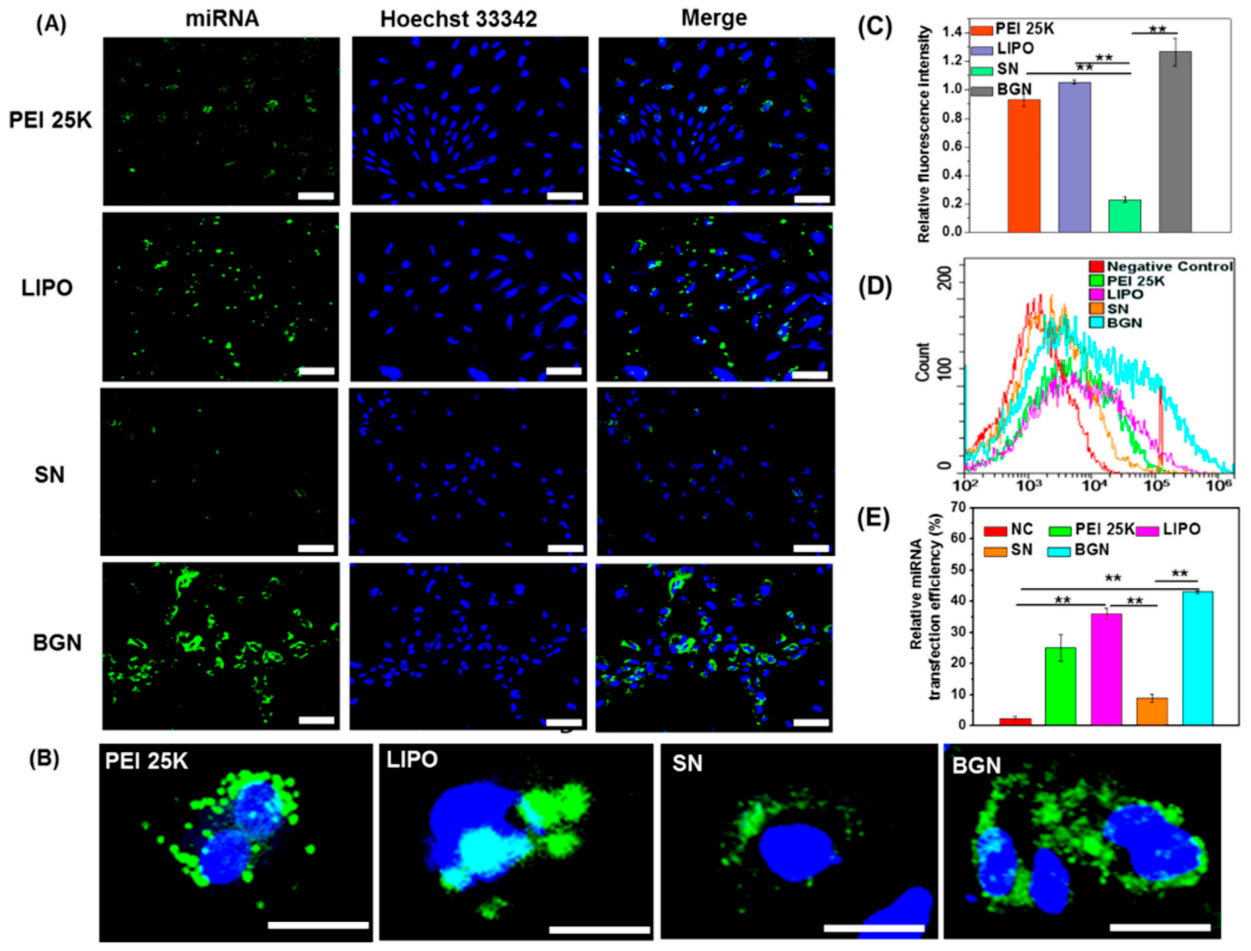
Transfection of FAM-labeled miRNA (0.26 mg/mL) into BMSCs with BGN (10 mg/mL), SN (10 mg/mL), and commercial PEI 25K and LIPO. (**A**) Fluorescent images of BMSCs, scale bar: 200 μm; (**B**) Magnified fluorescent images, scale bar: 50 μm; (**C**) Quantitative anaslysis of fluorescent signals in the cells; (**D**) Flow cytometry analysis of BMSCs; (**E**) Quantitative analysis of flow cytometry results. Adapated from reference [61] with permission. ** *p* < 0.01.

Meanwhile, due to the positive charges of miRNA binding peptide (KKKKKKKK), negatively charged miRNA could be efficiently complexed with the peptide through electrostatic and hydrophobic interactions. Using the bi-functional peptide as the linker to self-assmble MSNs with miRNA, miR-29b-MSN complexes were then prepared. With the complexes, miR-29b (25 nM, miRNA/MSN: 1.5/100 *w*/*w*) was successfully delivered by MSNs into NRK cells and protein expression levels of miR-29b targets were remarkably down-regulated by delivered miR-29b. In order to avoid using cationic peptides or polymers to complex siRNAs or miRNAs, Lei et al. developed calcium-doped silica nanoparticles (bioactive glass nanoparticles, or BGNs), which could bind with miRNA through the strong interaction between calcium and phosphate groups in miRNAs [61]. Upon packaging miRNA with the synthesized vectors, they found BGNs exhibited a 7-fold enhancement in miRNA binding, in comparison with normal silica nanoparticles (SNs). With FAM-labeled miRNA as probe, the transfection efficiency was then compared through BGNs, SNs, and commercial PEI 25K and lipofectamine (LIPO). As shown in Figure 5, transfection efficiency by BGNs was much higher than that by other vectors, demonstrating the strong binding between BGNs and miRNAs and the promise of BGNs as carriers for miRNA delivery. Additionally, even though MSNs demonstrated their ability to transport nucleic acids into cells, one major issue accompanying with the delivery by MSNs is the severe endosomal entrapment after endocytosis into cells, which could induce poor cytoplasmic delivery efficiency of nucleic acids and lead to subsequent poor gene silencing efficiency [75]. To address this issue, Yu et al. utilized the pore of MSNs, which was used to encapsulate choloroquine that could facilitate endosomal escape [64]. Upon delivery into endosome/lysosome, choloroquine was released to break the membrane of endosome/lysosome and siRNA was subseqeuntly released into cytoplasm. Using siRNA against PLK that can induce cell apoptosis through regulation of PLK, they compared the biological function of siRNA (100 nM, siRNA/choloroquine/MSN: 1/1/10 *w*/*w*/*w*) delivered by MSNs with or without choloroquine through measruing cell viability, demonstrating the presence of choloroquine could faciliate the delivery of siRNA into cytoplasm to exert its biological function.

**Figure 6:**
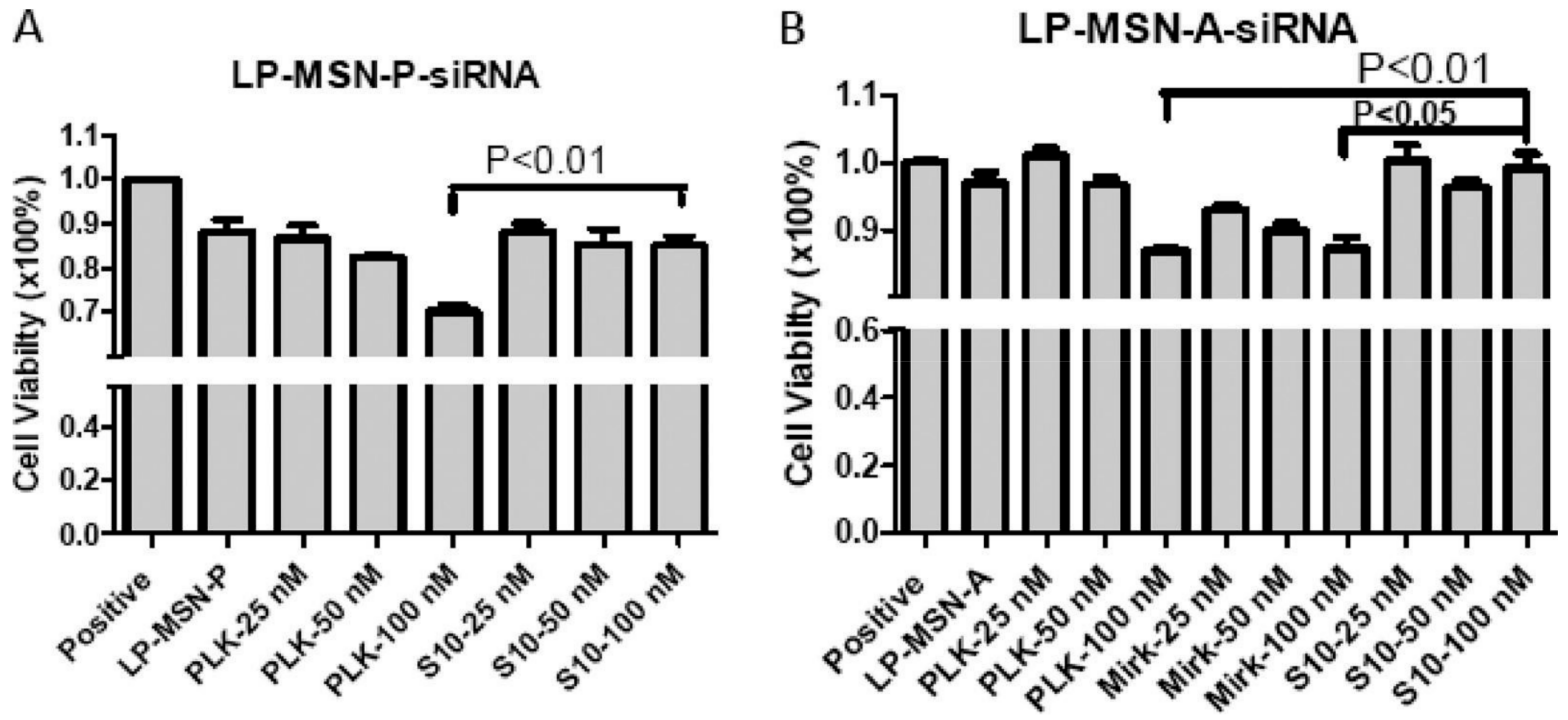
Dose-dependent effect of siRNAs against polo-like kinase 1 (PLK) and minibrain-related kinase (MirK) on viability of osteosarcoma cancer cell line KHOS cells, which were delivered by (**A**) LP-MSN-P or (**B**) LP-MSN-A. S10 that did not target PLK or MirK was used as negative control. Adapted from reference [39] with permission.

**Figure 7:**
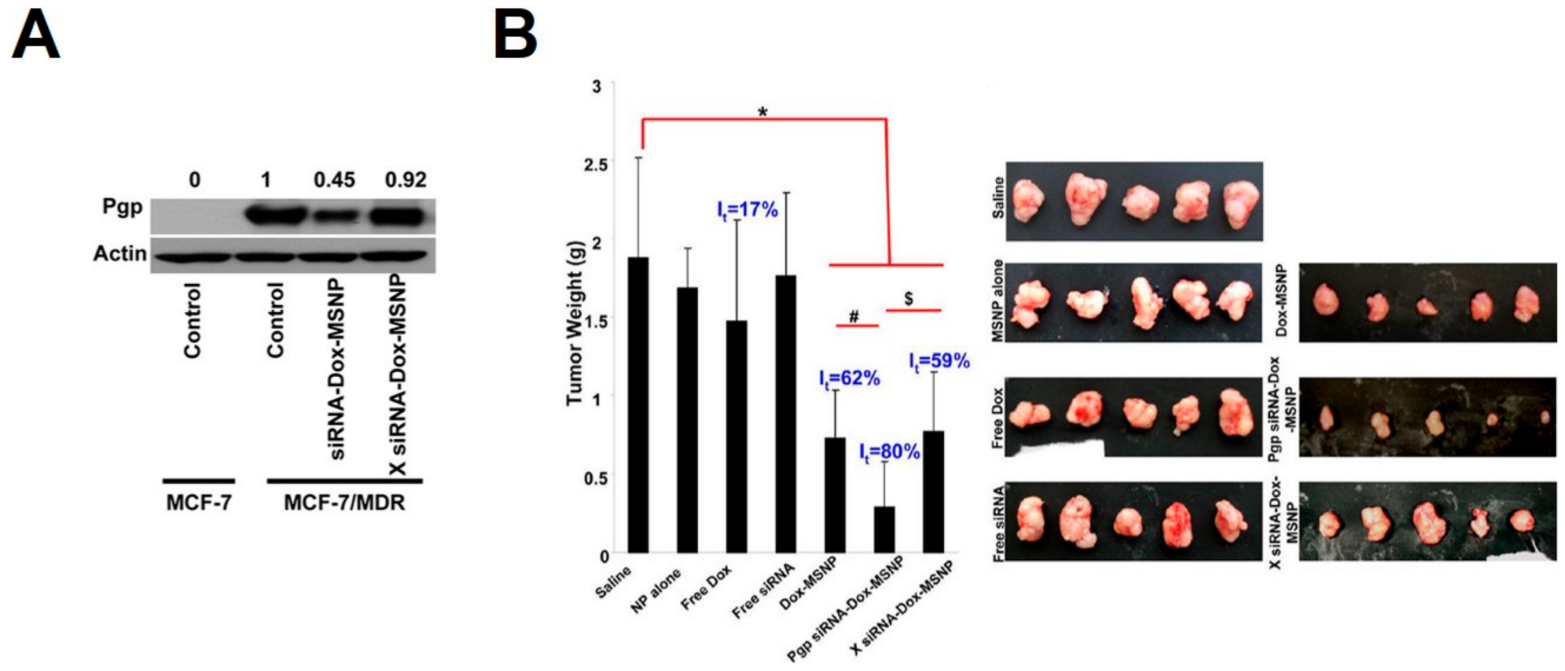
(**A**) Western blotting analysis of Pgp in MCF-7 cells or drug resistant MCF-7 cells post treatment with Pgp siRNA-Dox-MSNP or X siRNA-Dox-MSNP. SiRNA, 125 ng/mL; Dox, 0.4 μg/mL; MSNP, 12.5 μg/mL; (**B**) Sizes of tumors from drug resistant MCF-7 tumor bearing mice post treatment with control, free Dox, free siRNA, Dox-MSNP, Pgp siRNA-Dox-MSNP or X siRNA-Dox-MSNP. X siRNA indicates negative control RNAs. SiRNA, 0.024 mg; Dox, 0.08 mg; MSNP, 2.4 mg. Adapted from reference [69] with permission. * *p* < 0.05, relative to saline group; ^#^ *p* < 0.05, relative to Dox-MSNP group; ^$^ *p* < 0.05, relative to X siRNA-Dox-MSNP group.

With the successful delivery of siRNA or miRNA into cytoplasm, they were able to down-regulate target gene expression to realize therapeutic effects. For instances, tumor-suppressive miR-34a that has entered clinics was selected as the pro-drug for therapeutic treatments. Using MSNs as carriers, miR-34a was encapsulated by MSNs and an antibody targeting the cell surface antigen disialoganglioside GD2 (GD_2_) was conjugated onto the surface of MSNs for selective delivery [59]. By this miRNA-MSN complex, Davidoff et al. showed miR-34a (100 μg, miRNA/MSN: 1/10 *w*/*w*) was successfully delivered into neuroblastoma tumors in a murine orthotopic xenograft model, leading to a significant increase in miR-34a level and down-regulation in protein levels of miR-34a targets. Consequently, significantly decreased growth of neuroblastoma tumor was detected and the apoptosis in tumors was increased. In addition to using tumor-suppressive miRNAs as pro-drugs, siRNA pro-drugs targeting endogenous oncogenes were also designed and different siRNA-MSN complexes have been prepared for siRNA-guided therapy. Qiao et al. selected ongoneic minibrain-related kinase (MirK) and polo-like kinase 1 (PLK) as the therapeutic targets and used polylysine-functionalized large pore MSNs (LP-MSN-P) as delivery vectors to deliver siRNA (siRNA/MSN: 1/20 *w*/*w*) against them into osteosarcoma cancer cells [39]. Upon delivery into cells, these siRNAs consequently led to down-regulation of target expressions and dose-dependent reduction in cellular viability (Figure 6). Meanwhile, they found the delivery efficiency by LP-MSN-P was higher than that by amine-functionalized large pore MSNs (LP-MSN-A) (Figure 6). Human epidermal growth factor receptor 2 (HER2) is identified as an oncogene in breast cancers and also validated as the therapeutic target for treatment of breast cancers. Using MSNs as the carriers, siRNAs designed to knock down HER2 and an anti-HER2 antibody were then loaded onto MSNs to realize selective delivery of siRNAs into HER2-positive breast cancer cells rather than HER2-negative cells [65]. The successful delivery of siRNA (60 nM, siRNA/MSN: 1/50 *w*/*w*) then led to significant and selective reduction in cellular viability. Similarly, vascular endothelial growth factor (VEGF) is an oncogene and siRNAs against VEGF (3.5 nmol, siRNA/MSN: 1/40 *w*/*w*) were then designed and delivered into tumor bearing mice by using MSNs as carriers [66,67]. The growth of A549 tumors were monitored post treatment, showing delayed growth of tumor treated with siRNA-MSN complexes rather than with control treatments. Noteworthily, siRNA against VEGF (3.5 nmol, siRNA/MSN: 1/40 *w*/*w*) was also systematically administrated into orthotropic ovarian tumor-bearing mice with MSNs as carriers, showing significant inhibition of angiogenesis and retardation of tumor growth [68]. On the other hand, MSN-facilitated delivery of siRNA was also able to overcome chemodrug resistance and to realize enhanced therapeutics. For example, Nel et al. selected the drug expoter, P-glycoprotein (Pgp) that is over-expressed in drug-resistant cancer cells (Figure 7A), as the siRNA target and used MSNs to load Pgp siRNA and small-molecular drug doxorubicin (Dox) [69]. Upon delivery of Pgp siRNA into drug-resistant breast cancer cells by MSNs, Pgp levels were significantly down-regulated by the delivered Pgp siRNA (Figure 7A). When treatment of tumor-bearing mice with the MSN-siRNA-Dox complexes, tumor growth was also reamarkbly inhibited (Figure 7B). In comparision with Dox-MSN complexes, control siRNA-Dox-MSN complexes did not show improved therapeutic efficiency, demonstrating the down-regulation of Pgp by MSN-delivered siRNA was essential for overcoming the drug resistance of cancer cells.

### 3.3. ASO-MSN Complexes for ASO Delivery and ASO-Guided Therapeutics

Since ASOs are able to inhibit target gene expression and hold great potential in therapeutics, ASOs have also been delivered into cells to suppress target genes through preparation of ASO-MSN complexes for facilitating ASO delivery. Simiarly as DNA and miRNA/siRNA, the delivery of ASO into cells also depends on the endocytosis of ASO-MSN complexes and release of ASO from MSNs inside cells. For instance, a charge-neutral peptide nucleic acid (PNA), which is one of the ASOs against B-cell lymphoma 2 (Bcl-2) and can not be attached onto the surface of MSNs through electrostatic interactions, was covalently conjugated to the surface of MSNs through disulfide bond [70]. Post treatment of cells with PNA-MSN complexes, the complexes were efficiently delivered into cells and PNA was released from MSNs upon cleavage of disulfide bond by biomolecules inside cells.

**Figure 8:**
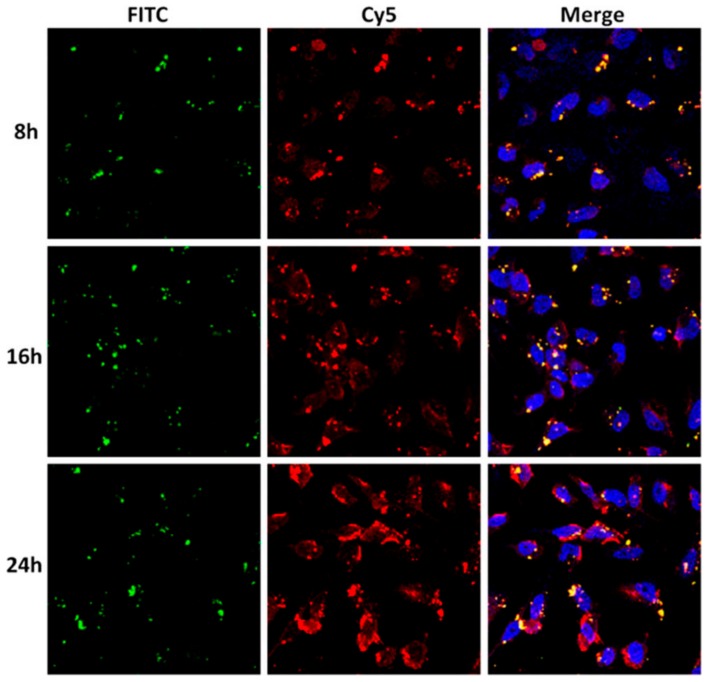
Time-dependent trafficking of the delivery and release processes of PNA delivered by MSNs into HeLa cells. Cy5 and FITC were used to label PNA and MSNs, respectively. PNA, 0.2 μM; PNA/MSN, 1/20 (*w/w*). Adapted from reference [70] with permission.

**Figure 9:**
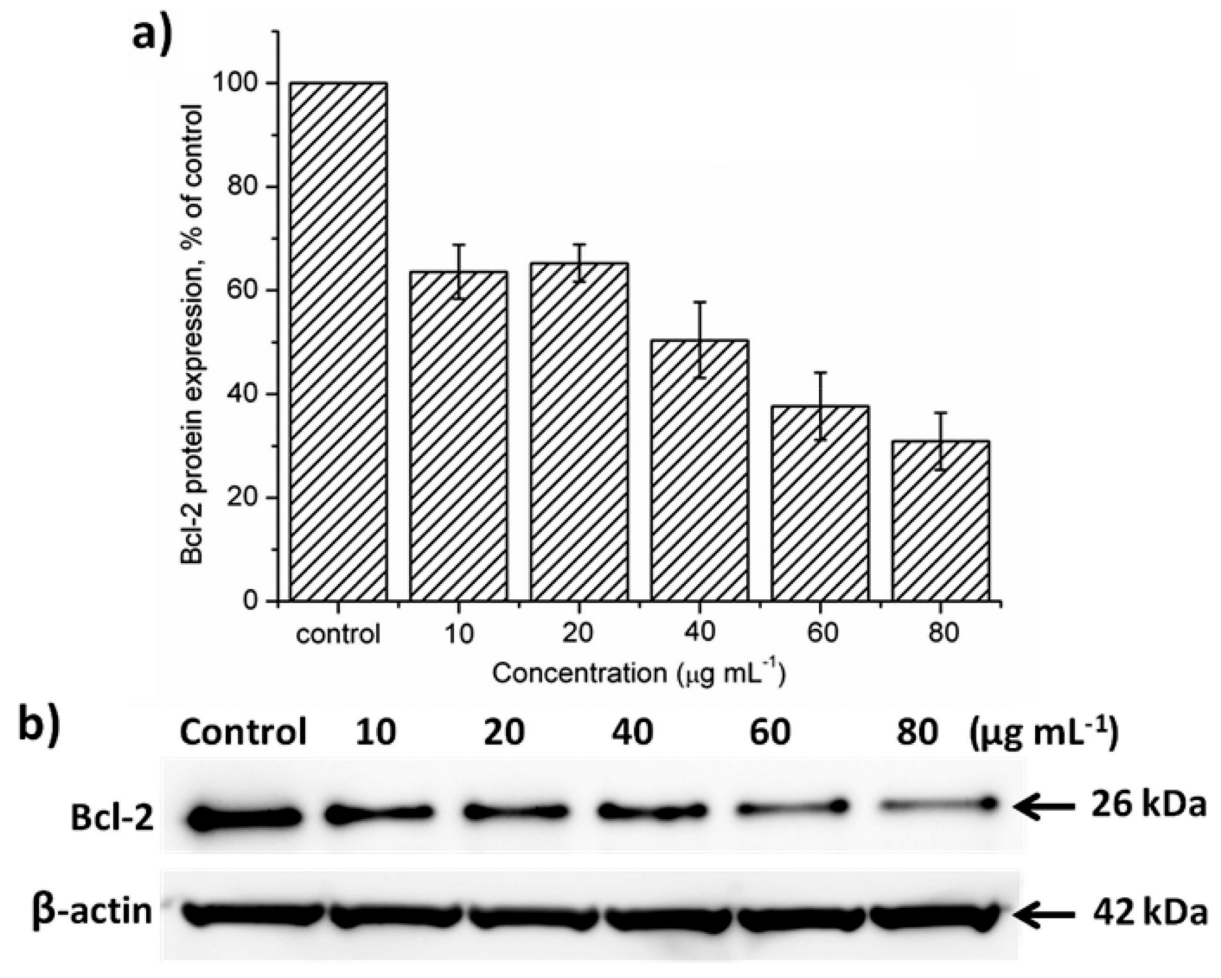
Analysis on protein expression levels of Bcl-2 after delivery of PNA into HeLa cells. (**a**) Quantitative analysis and (**b**) western blotting results of Bcl-2 protein expression levels. PNA/MSN, 1/20 (*w/w*). Adapted from reference [70] with permission.

Through labeling PNA with Cy5 and MSNs with FITC, the delivery and release processes were monitored in real-time under confocal microscope, showing time-dependent delivery of PNA-MSN complexes inito HeLa cells and release of PNA from MSNs into cytoplasm (Figure 8). Consequently, with the successful delivery of PNA by MSNs into cytoplasm, the protein expression levels of Bcl-2 in HeLa cells were remarkbly down-regulated by PNA as expected (Figure 9).

**Figure 10:**
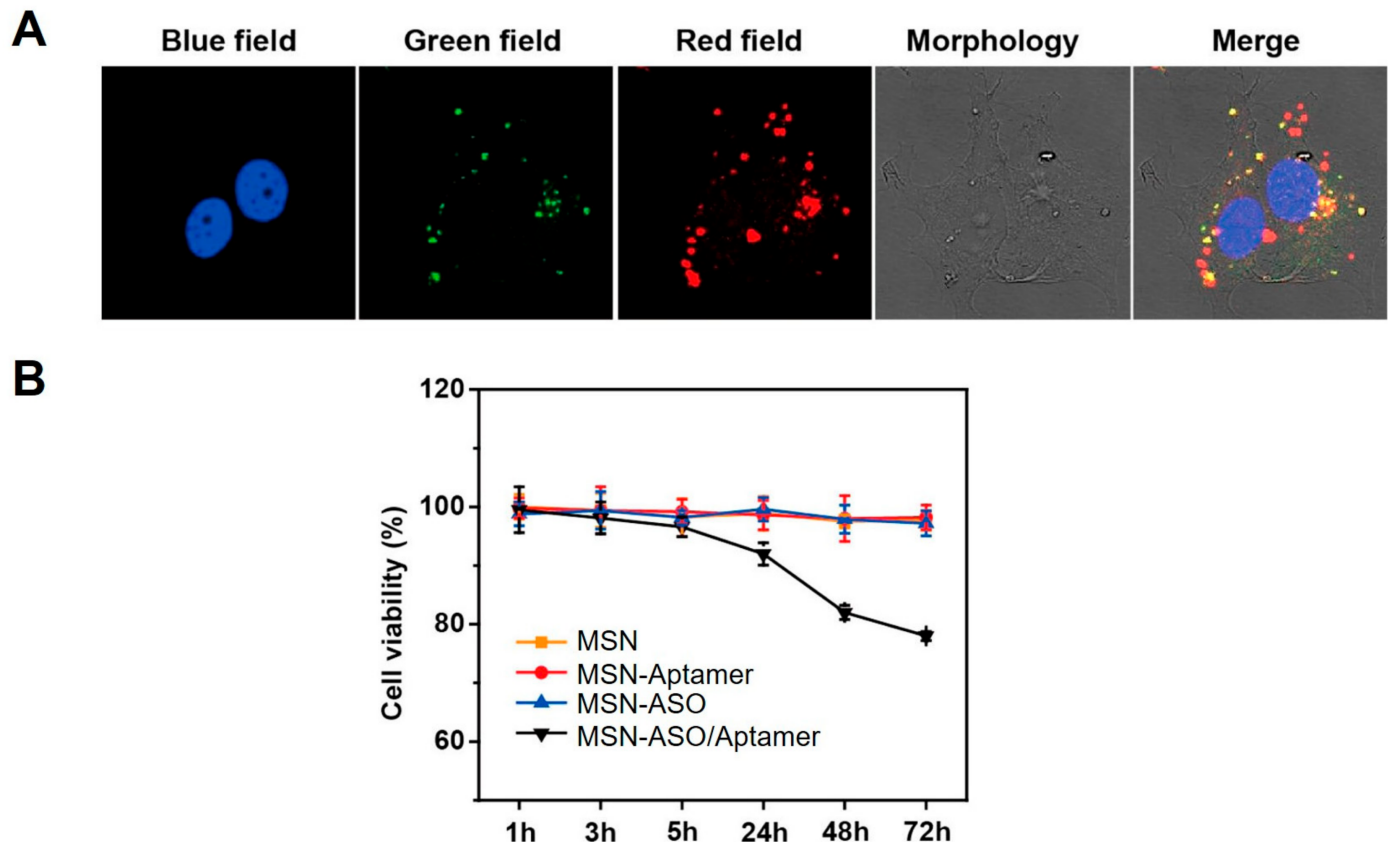
(**A**) Confocal fluorescent images of MCF-7 cells post treatment with MSN-ASO/Aptamer. Blue field, nucleus staining. Green field, FAM-labeled ASO. Red field, MSN fluorescence; (**B**) Cellular viability of MCF-7 cells post treatment with MSN, MSN-aptamer, MSN-ASO, or MSN-ASO/Aptamer. ASO, 50 nM; MSN, 100 μg/mL. Adapted from reference [71] with permission.

On the other hand, ASOs could target not only endogenous mRNAs but also endogenous miRNAs. With the delivery of ASO by ASO-MSN complexes, the specific inhibitory functions of ASOs have also been used to achieve multiple functions in miRNA-related biomedical applications. For example, to realize simultaneous inhibition and imaging of miR-21 inside cells, dual-functional and double-stranded ASOs were synthesized and delivered through packaging with MSNs. The double-stranded ASOs contained a strand of FAM-labeled ASO that targets endogenous miR-21 and a short complementary strand for this ASO that were labeled with DABCYL. Initially, the fluorescence of the double-stranded ASOs was quenched due to the fluorescence resonance energy transfer (FRET) effect between FAM with DABCYL, which could be recovered upon binding of ASO with miR-21 inside cells to remove the strand with DABCYL away from ASO [71]. The double-stranded ASOs were then attached onto MSNs through disulfide bond and MSNs were also modified with aptamer to achieve target-selective delivery. Upon treatment of MCF-7 cells that overexpress miR-21 with the ASO-MSN complexes, significant fluorescence turn-on was observed (Figure 10A), indicating successful binding of ASO with miR-21 inside cells. Meanwhile, the inhibition of miR-21, which is an oncogenic miRNA in cancer cells, by the delivered ASOs also led to reduced cellular viability (Figure 10B).

Utilizing the specific property that MSN pores could encapsulate small molecule drugs and ASOs could not only bind with miRNA, but also cap the pore of MSNs, small molecule drugs that could regulate miRNAs and ASOs that could inhibit the same miRNAs were simultaneously delivered into cells by MSNs to realize enhanced biological functions [72,73]. Upon delivery of small molecule-ASO-MSN complexes into the cells, due to the capping of the pore with ASOs that prevent small molecules from entering cytoplasm, small molecules inside the pore initially could not inhibit miRNA, while, the binding of ASOs with endogenous miRNAs then uncapped the pore and released the encapsulated small molecules into the cytoplasm, realizing dual-inhibition of miRNAs. Using this method, Yao et al. demonstrated the dual-inhibition of miR-122 (MSN, 25 μg/mL; antagomir-122, 200 nM; small molecule, 2 μM) in hepatocellular carcinoma cells [72] and miR-21 (MSN, 50 μg/mL; antagomir-122, 150 nM; small molecule, 4.5 μM) in ovarian cancer cells [73]. In light of that miR-21 functions as an oncogene in ovarian cancer cells and inhibition of miR-21 has been shown as an effective way to treat ovarian cancers, small molecules and ASOs delivered by MSNs that could inhibit miR-21 in HeLa cells then significantly promoted cellular apoptosis [73].

## 4. Perspective 

Biocompatible MSNs have been widely used as biomaterials for the delivery of different cargos into biological systems and achieve diverse biological functions. In this review paper, we mainly introduced the applications of MSNs in nucleic acid delivery and nucleic acid-guided therapeutics. While MSNs have been shown as effective carriers for intracellular delivery of nucleic acids, one major issue accompanying the delivery was the endosomal entrapment, which led to poor cytoplasmic delivery efficiency and subsequent impaired biological function of the delivered nucleic acids. While encapsulation of chloroquine inside MSNs could facilitate endosomal escape upon delivery into cells [64,76], the cytotoxicity of chloroquine then resulted in another problem for delivery. Biocompatible peptides that also can break the endosome membrane may serve as possible endosomal escape agents [77]. Besides, full utilization of the large pore of MSNs may contribute to various other biomedical applications. For example, “on-demand” release could be realized through using molecules to cap the pores that encapsulated other functional molecules [78,79,80,81]. In addition, even though nucleic acids could exert their biological function upon delivery into cells and subsequent therapeutic outcomes could be detected, combined use with chemotherapeutic drugs has been shown to be another promising strategy for therapeutic treatments. The co-delivery of siRNA with doxorubicin was able to overcome drug resistance in breast cancer both in vitro and in vivo [69,82]. Enhanced therapeutic effects could also be achieved by co-delivery of nucleic acids with other drugs [83,84,85]. With the development of MSNs modified with multiple functionalities or combined use with other functional biomolecules, MSNs will find more applications in drug delivery, therapeutics and even therapeutic evaluations in the future.

## Figures and Tables

**Table 1:** Properties of MSNs and sequences of short oligonucleotides mentioned in this section.

MSNs			Nucleic Acids/Fluorophore-Labeled Nucleic Acids			Ref.
MSN Size (nm)	Pore Size (nm)	Surface Coatings	Name	Antisense Strand (5’-3’)	Sense Strand (5’-3’)	
~200	~2.71	Carbosilane dendrons	ssDNA^Cy3^	Cy3-TTATCGCTGATTCAAGACTGA	/	[52]
~180	/	l-histidine	pDNA-Luciferase	/	/	[53]
~120	~2.5	PEI	pDNA-GFP	/	/	[37]
~70	~3.5–5	Amine with disulfide linker	dsDNA	CTGTGGTTGTGTTTGCACTTT	AGTGCAAACACAACCACAGTT	[54]
~80	~2.6	Poly-arginine	pDNA-mCherry	/	/	[40]
~250	~23	Amine	pDNA-GFP	/	/	[55]
~130	~12	Amine	dsDNA	TCAGAGAGTTAGAGAGTTAGAGAGTCAGAGAGTTAGAGAGTTAGAGAGTCAGAGAGTTAGAGAGTTAGAGAG	CTCTCTAACTCTCTAACTCTCTGACTCTC-TAACTCTCTAACTCTCTGACTCTCTAACTCTCTAACTCTCTGA	[56]
~150	/	Amine	pDNA-HNF3β	/	/	[46]
~205	~2.64	Amine	pDNA-BMP2	/	/	[57]
~200	~3	Amine	pDNA-PDGF	/	/	[58]
~74	/	Poly-amidoamine	miR-34a	UGGCAGUGUCUUAGCUGGUUGU	CAAUCAGCAAGUAUACUGCCCU	[59]
~219.5	~18.8	Bifunctional peptide	miR-29b	GCUGGUUUCAUAUGGUGGUUUAGA	GCUGGUUUCAUAUGGUGGUUUAGA	[60]
~250	~3.5	Calcium	miR-5106	AGGUCUGUAGCUCAGUUGGCAGA-FAM	UCUGCCAACUGAGCUACAGACCU	[61]
~150	~10	PDMAEMA	siRNA-PLK	UUAAGCAGCUCGUUAAUGGTT-FAM or Cy3	CCAUUAACGAGCUGCUUAATT	[62]
~130	~2.5	PEI	siRNA-Kras	UUUCCUACUAGGACCAUAGGT	CUAUGGUCCUAGUAGGAAATT	[63]
~100–200	~28	Polylysine	siRNA-MirK	UUCCGGAACAUGAAGUGCCGC	GGCACUUCAUGUUCCGGAAUU	[39]
~100–200	~28	Polylysine	siRNA-S10	ACGUCGCAGUAACUGUUGCUU	ACGUCGCAGUAACUGUUGCUU	[39]
~100–200	~11	PDMAEA	siRNA-PLK	UUAAGCAGCUCGUUAAUGGTT	CCAUUAACGAGCUGCUUAATT	[64]
~100	/	PEI	siRNA-HER2	UUGGGCAUGGACUCAAACGUGUU	CACGUUUGAGUCCAUGCCCAAUU	[65]
~50	~3.6	PEI	siRNA-VEGF	GAUCUCAUCAGGGUACUCCTT	GGAGUACCCUGAUGAGAUCTT	[66,67,68]
~51	/	PEI	siRNA-Pgp	CGGAAGGCCUAAUGCCGAATT	UUGCGCAUUAGGCCUUCCGTT	[69]
~110	/	Amine with disulfide linker	PNA-Bcl2	TCTCCCAGCGTGCGCCAT-Cy5	/	[70]
~60	/	Amine	Double-stranded ASOs targeting miR-21	GTCAACATCAGTCTGATAAGCTATGTCGC-FAM	DABCYL-GCGACATAGCT	[71]
~120	~3	Amine	Antagomir-122	ACAAACACCATTGTCACACTCCA	/	[72]
~105	~3.2	Amines	Antagomir-21	UCAACAUCAGUCUGAUAAGCUA	/	[73]
